# Local responses to the threats of dramatic crises: do institutional leaders make a difference, and if so, how?

**DOI:** 10.1111/disa.70059

**Published:** 2026-05-15

**Authors:** Giliberto Capano, Alexandra D′Angelo

**Affiliations:** ^1^ University of Bologna Italy; ^2^ University of Padova Italy

**Keywords:** crisis response, decision making, Emilia‐Romagna Region, floods, local leaders

## Abstract

This study examines the complexity of disaster response management and the role of local institutional leadership. Drawing on a comparative analysis of the May 2023 floods in two Emilia‐Romagna provinces in Italy, it explores how local leadership influences the outcomes of crisis management within a shared institutional and hazard context. Using local press reports, municipal ordinances, and interviews with key figures, the study reconstructs the decision‐making processes involved and identifies critical leadership practices. The findings demonstrate that differences in preparedness, coordination, and post‐event trajectories are significantly impacted by the experience and professional background of leaders, as well as their capacity to make decisions in circumstances characterised by uncertainty and time pressure. By isolating leadership practices under constant structural conditions, the study makes a valuable contribution to disaster research by specifying how local agency shapes responses and learning in multi‐level disaster governance. It highlights, in particular, the importance of investing in local leadership development to strengthen municipal disaster management capacities.

**Practitioner points**

- Local leaders' prior experience of disaster management, epistemic orientation, and decision‐making styles are key drivers of leadership behaviours.
- Primary leadership behaviours include recognising early warning signs, taking precautionary measures, communicating effectively, and coordinating with other institutions.
- Improving crisis preparedness requires sustained investment in leadership development and training at the local level.

## 1 INTRODUCTION

The rising occurrence of complex modern crises, including disasters triggered by natural hazards, pandemics, and technological disruptions, makes local crisis management more essential than ever (Kapucu, 2008; Boin, Kuipers, and Overdijk, 2013). Central to such responses are institutional leaders, particularly those within local government. Although operating within national frameworks, local governments act as first responders, executing pre‐established plans and policies while also formulating grassroots disaster mitigation strategies (OECD, 2020). Thus, leadership at the local level is a vital factor in crisis management effectiveness, both during emergency response and throughout recovery (Christensen, Lægreid, and Rykkja, 2016; Ansell, Sørensen, and Torfing, 2021). Local institutional leaders, such as mayors, municipal executives, and agency heads, are responsible not only for coordinating immediate responses but also for sustaining public trust, mobilising communities, and managing in complex, often decentralised, institutional settings (Comfort, 2007; ’t Hart, Rosenthal, and Kouzmin, 1993).

Despite the critical involvement of local leadership in crisis contexts, few studies have examined this topic. While it is often assumed that these leaders play a key role in performing functions such as early detection, decision making, communication, and meaning making, empirical analyses remain limited (Jong, Dückers, and van der Velden, 2016a). Part of the ambiguity surrounding their engagement stems from how crises are typically handled in advanced democracies—through top‐down, institutionally designed civil protection systems (Alexander, 2002). Especially in dramatic crises, such as floods or pandemics, the coordination between vertical (national–local) and horizontal (inter‐agency) structures may obscure the contributions of local leadership (Boin and ’t Hart, 2010).

Nonetheless, crises are always, to some extent, local affairs. The actions—or inactions—of institutional leaders at the local level can shape outcomes decisively (Lalonde, 2007).

This article contributes to the field of disaster scholarship by isolating individual leadership agency through a controlled comparison. By examining two Italian provinces affected by catastrophic flooding in May 2023 and with highly similar institutional frameworks and exposure to hazards, but with different outcomes, we demonstrate that leadership behaviour, rather than structural conditions, determines crisis effectiveness. This design empirically reveals what the literature assumes but rarely shows: that individual characteristics (such as experience, epistemic orientation and decision‐making style) are decisive when contextual variables are constant. We advance disaster research by reconceptualising epistemic orientation as learnable institutional practices rather than psychological traits. We also provide a replicable analytical framework that identifies observable drivers of leadership effectiveness across disaster environments.

The paper is structured as follows. Section two reviews the literature and establishes our analytical framework for the assessment of leadership behaviour. Section three describes the research design and section four presents the empirical findings. Section five examines the theoretical and analytical implications which emerge from the empirical evidence presented. Section six offers some conclusions.

## 2 PUBLIC LEADERS AND CRISIS MANAGEMENT

### 2.1 Leadership in crisis management

The study of leadership in crisis management has evolved from early descriptive accounts towards a more nuanced body of theory, which recognises the central role of leadership in interpreting, governing and politically legitimising crises. In fields such as public administration, political science, organisational studies, and disaster scholarship, crises are now understood as socially mediated phenomena, rather than as exogenous shocks. In these contexts, authority, meaning, and responsibility are constructed dynamically (Boin et al., 2005). Consequently, leadership is not considered as epiphenomenal, but rather as constitutive of crisis governance itself.

Within public administration scholarship, crisis leadership encompasses strategic functions that extend beyond operational command. Pioneering research has identified primary responsibilities such as recognising threats, making sense of situations, taking decisions under time constraints, coordinating across boundaries, creating public narratives, and managing accountability (Boin et al., 2005). This literature demonstrates that crises expose the political and symbolic dimensions of governance, requiring leaders to stabilise expectations, protect institutional legitimacy, and navigate emergent blame dynamics (’t Hart, 2014).

Organisational theory has reframed crises as epistemic and cognitive challenges in the first instance. Key contributions to sensemaking theory argue that crises are characterised by radical ambiguity, disrupted causal assumptions, and collapsed routines (Weick, 1988, 2010). Leadership becomes intrinsically linked to interpretive processes through which events acquire meaning and actionability. Leaders act as pivotal figures in constructing plausible narratives of evolving situations, arbitrating between competing expert knowledge, and directing organisations towards courses of action that retroactively stabilise meaning (Maitlis and Sonenshein, 2010).

Research into strategic leadership shows that crises amplify leaders' experiential histories, cognitive orientations, and behavioural repertoires rather than their formal authority (Schaedler, Graf‐Vlachy, and König, 2022). They act as ‘revealing moments’, exposing differences in judgement, tolerance of ambiguity, and ability to mobilise resources (Boin and Lodge, 2016).

Disaster governance scholarship has embedded these insights in multi‐level governance architectures. This literature documents how crisis leadership unfolds across interdependent institutional arenas, necessitating vertical coordination with higher governmental tiers and horizontal collaboration with non‐state actors, epistemic communities, and civil society (Kapucu, 2008; Kapucu and Ustun, 2018). Rather than functioning as autonomous decision‐makers, leaders act as brokers of authority, information, and legitimacy within polycentric systems.

Across these traditions, a shared insight emerges: crisis leadership is both highly consequential and markedly variable. Although functional imperatives recur, leaders interpret risk differently, make decisions in distinct sequences, communicate publicly in various ways, and balance technical, political, and normative considerations in distinct ways (Christensen, Lægreid, and Rykkja, 2016). Leadership effectiveness depends on situational pressures, relational dynamics, and institutional contexts, rather than on universal prescriptions.

Crisis leadership is thus a multidimensional phenomenon, integrating strategic, cognitive, communicative, and political functions. It is an interpretive practice that transforms uncertainty into actionable frameworks, enabling coordination under pressure and maintaining legitimacy amid disruption. While these theoretical traditions establish that crisis leadership is consequential yet variable, a systematic understanding of what drives this variability, particularly at the local level, remains limited. This paper addresses the gap by examining the behavioural drivers that shape how local institutional leaders respond to crises.

### 2.2 The role of local leaders in crisis management

Local institutional leaders can play a pivotal role in crisis management by offering context‐specific insight, fostering collaboration, and ensuring rapid, tailored responses. Yet, the effectiveness of this leadership varies considerably: while some leaders successfully navigate crises and strengthen community resilience, others fail to respond adequately, exacerbating harm and eroding public trust. Their leadership is essential for aligning local realities with broader emergency strategies and cultivating resilience within their communities (Alexander, 2015).

Three interconnected factors explain why local leadership proves so decisive in crisis outcomes. First, local leaders possess in‐depth knowledge of the social, cultural, and economic characteristics of their community. Their understanding enables them to develop flexible crisis responses tailored to their local context. Margoński (2018) emphasises that local leaders play a crucial part during crises because they create stability, which decreases uncertainty and stress among both employees and residents. Local leaders demonstrate better capabilities than centralised authorities to detect upcoming needs and execute rapid responses while effectively utilising local resources.

Second, successful crisis governance depends heavily on the interpersonal and communication abilities of local leaders. Public anxiety declines when officials provide transparent and timely information through empathetic communication, which helps people to follow emergency measures. According to Samad, Jerjawi, and Dadich (2023), effective political leaders demonstrate empathy, ethical conduct, and resilience when handling crises. Through these characteristics, local leaders establish trust, which makes them reliable connectors of public and higher government entities (Ansell and Boin, 2019).

Third, the ability of local leaders to coordinate with stakeholders is a decisive factor in the success of crisis response systems. Leaders bridge the gap between top‐down policies and grassroots realities by acting as integrators of local resources. Eid et al. (2023) note that effective local leadership involves both immediate responsiveness and long‐term strategic learning.

Local leaders utilise social capital, such as community networks, trust, and shared norms, to enhance resource mobilisation and recovery (Kapucu, 2008; Norris et al., 2008). This serves both immediate emergency management and longer‐term community resilience (Margoński, 2018; Eid et al., 2023; Samad, Jerjawi, and Dadich, 2023).

These principles are particularly evident in mayoral leadership. Mayors are central figures in urban crisis management, with responsibilities that range from immediate emergency response to long‐term community recovery. Their leadership has proven vital in recent crises, with examples of both commendable action and notable shortcomings. During the COVID‐19 (coronavirus disease 2019) pandemic, several mayors in the United States adopted proactive, community‐centred approaches. For instance, Mayor London Breed of San Francisco, California, issued an early shelter‐in‐place order, which helped to reduce the spread of the virus (Duane, 2020). In the United Kingdom, Mayor of London Sadiq Khan emphasised public transport safety and housing support during the pandemic (Washington Post Live, 2021). Dutch mayors responded to the Malaysia Airlines Flight 17 tragedy in 2014 by coordinating memorials and offering community support (Jong, Dückers, and van der Velden, 2016b), whereas in the Czech Republic, local authorities managed flood responses effectively through planning and volunteer mobilisation (Bera, 2023). Mayor Carmen Yulín Cruz of San Juan, Puerto Rico, was praised for her leadership during Hurricane Maria in 2017 (Giles, Howitt, and Leonard, 2021). During the 2009 Red River floods, Mayor Dennis Walaker of Fargo, North Dakota, balanced community mobilisation with psychological well‐being by encouraging residents to ‘take a break’ despite worsening forecasts (O'Neill et al., 2016, p. 75). Similarly, research on Thai municipalities following floods in 2011 shows that local government leaders' disaster resilience abilities had statistically significant positive effects on flood management progress (Khunwishit, Choosuk, and Webb, 2018), while studies of coastal cities of Florida reveal that local government managers' hazard‐specific risk perceptions significantly influence resilience planning implementation even after controlling for objective risks (Kim et al., 2024).

Conversely, other mayoral responses have attracted significant criticism. Water contamination issues in Flint, Michigan, remained unaddressed by Mayor Dayne Walling (Hanna‐Attisha et al., 2016) and Mayor Ray Nagin's response to Hurricane Katrina in 2005 received intense public criticism (Roberts, 2006). The waste management crisis in Naples, Italy, and failed sanitation reform in Bogotá, Colombia, also reflect governance challenges (Armiero and D'Alisa, 2012; Gallini, 2016). And in New York City, New York, Mayor Bill de Blasio's early minimisation of COVID‐19 hindered timely action (Goodman, 2020).

These examples illustrate mayors' centrality in crisis management, yet their effectiveness is shaped by varying institutional mandates, governance structures, and local capacities—factors examined in the following subsection.

### 2.3 Theoretical framework: key drivers of local leaders' behaviour vis‐à‐vis decision making under pressure

Local leaders must overcome significant obstacles when making crisis decisions, as they need to deliver swift and strategic actions. The COVID‐19 pandemic, together with disasters triggered by natural hazards and outbreaks of Ebola virus disease, show that local choices determine what happens to communities. Empirical research on flood governance reveals that local leaders' capacity to interpret risk forecasts and implement precautionary measures significantly influences disaster outcomes, with measurable variations in effectiveness across municipalities facing identical hazards (Khunwishit, Choosuk, and Webb, 2018; Slavíková, Raška, and Kopáček, 2019). Leaders in crisis situations face additional challenges because they must make decisions quickly while working with limited information and enduring intense public scrutiny. Liu and Christensen (2022) emphasise that local leaders must make prompt decisions based on accurate information when confronted with unpredictable situations.

Emotional awareness is also demanded by these decisions, aligning with the findings of Skagerström et al. (2023) on the importance of adaptive and empathetic leadership. Building trust is essential. Effective leaders demonstrate transparency and emotional intelligence, reassuring communities under stress (Scarpis et al., 2023). Additionally, collaborative decision making is vital. Hadley et al. (2011) underscore that incorporation of diverse stakeholder perspectives enhances crisis response effectiveness. Leaders can coordinate with agencies, non‐profit organisations, and community groups. This not only allows them to pool resources, but also to create unified strategies. Engaging citizens further augments contextual understanding and responsiveness (Hadna, 2022). Lastly, and above all, local institutional leaders take critical decisions that can make the difference (Bera, 2023).

While local institutional leaders are central actors in crisis management, the drivers of their concrete actions are still under discussion. Certainly, their behaviour is not only determined by institutional mandates and formal roles. Many studies have shown that various factors, when combined, can explain the behaviour of local leaders when it comes to dealing with crisis management and critical situations (Wu et al., 2021; Bera, 2023; Chen and Liu, 2026). Drawing on this literature, we suggest that the interaction of contextual conditions, personal experience, epistemic orientation, and decision‐making style shapes leaders' behaviour.

#### 2.3.1 Contextual factors

Local governance systems can differ significantly in structure, capacity, and political and institutional practices. These contextual variables have a strong influence on leadership behaviour during crises. In centralised systems, mayors or local executives may function primarily as implementers of national policies, whereas decentralised systems afford them greater discretion to craft local responses (Christensen, Lægreid, and Rykkja, 2016; OECD, 2020). The degree of municipal autonomy has been linked to adaptive capacity and responsiveness (Ladner and Soguel, 2015).

Moreover, the administrative resources and institutional preparedness of local governments also matter. Well‐established risk management plans and inter‐agency coordination mechanisms in municipalities enable leaders to act more decisively and efficiently (Comfort, 2007; Kapucu, 2008). Community‐level variables such as citizen engagement, social capital, and local trust in institutions can further facilitate or constrain crisis responses (Norris et al., 2008; Berkes and Ross, 2013).

#### 2.3.2 Prior experience of leaders

Experience of emergencies can profoundly shape how local leaders respond under pressure. Those who have navigated past crises often possess operational knowledge, stronger networks, and enhanced situational awareness (Boin, Kuipers, and Overdijk, 2013; Jong, Dückers, and van der Velden, 2016a). Prior exposure to high‐stakes decision making tends to reduce hesitation and improve coordination (Cigler, 2007). Margoński (2018) emphasises that experienced local leaders can instil confidence in residents and staff by projecting calmness and authority. Moreover, leaders with previous reform or governance experience may be better equipped to recognise systemic weaknesses and initiate structural improvements during or after crises (Helsloot and Ruitenberg, 2004).

#### 2.3.3 Epistemic orientation

The most recent major global crisis, the COVID‐19 pandemic, once again highlighted the importance and complexity of leaders engaging with scientific expertise. Crises often involve contested, incomplete, or rapidly evolving scientific knowledge, requiring leaders to navigate uncertainty and conflicting expert opinions (Weible et al., 2020). Effective crisis leadership necessitates thorough engagement with the available scientific evidence, while also acknowledging its limitations and uncertainties. Simply following the science is not enough: leaders who transparently communicate the basis for their decisions, including which evidence they prioritise and why, tend to foster greater public trust than those who present scientific consensus as unambiguous when it is not (Christensen and Lægreid, 2020; Ansell, Sørensen, and Torfing, 2021). This epistemic orientation reflects a leader's willingness to base decisions on the best available evidence while being transparent about the inherent uncertainties of crisis situations (Boin and ’t Hart, 2010).

#### 2.3.4 Decision‐making style

A leader's decision‐making style—whether intuitive or analytical, centralised, or participatory—also determines crisis outcomes. In this study, we are less interested in the various dimensions of leadership proposed in the literature, such as transformative and transactional, or collaborative (Bass and Riggio, 2006; Ansell and Gash, 2008; Kapucu and Garayev, 2011), than in how they approach decisions. Some leaders operating in crises may rely on improvisational and intuitive decision making, whereas others may adopt a structured approach based on scenario planning and cross‐agency consultations (Boin et al., 2005; Lalonde, 2007). Above all, however, they must decide how to proceed. Among the styles recognised in the literature, a critical dichotomy lies between proactive and routine/passive decision making.

Proactive decision making refers to anticipatory, future‐oriented actions that involve initiating change, identifying opportunities, and mitigating risks before they become urgent (Bateman and Crant, 1993). Proactive leaders are characterised by strategic foresight, creativity, and an inclination towards innovation and learning. This style is closely tied to entrepreneurial behaviour, adaptive leadership, and transformational leadership theories (Bass, 1985; Yukl, 2008). Bateman and Crant (1993) define a proactive personality as the disposition to effect environmental change rather than merely respond to it. This idea is extended in Parker and Collins (2010), who distinguish between proactive work behaviour and more reactive, compliance‐driven actions.

In contrast, routine or passive decision making is characterised by adherence to established procedures, reactions to problems as they arise, and an overall preference for stability and predictability. This style aligns with Weberian bureaucratic rationality and satisficing behaviour under bounded rationality, as discussed by March and Simon (1958). Routinary decision‐makers often avoid change and prefer to work within known systems and processes. They primarily react to external stimuli, such as regulatory changes, crises, and stakeholder pressure, rather than taking proactive measures. The approach helps decision‐makers to save resources and operate efficiently, but it restricts their ability to innovate and adapt. The ‘threat rigidity’ model developed by Staw, Sandelands, and Dutton (1981) shows that people and organisations tend to stick with familiar patterns when facing stress or uncertainty, even though proactive action would be more suitable. This framework suggests that legitimacy stems from formal correctness rather than response effectiveness, as decision‐makers prioritise procedural safety over innovative solutions. These approaches fall within the dynamics of ‘blame avoidance’, which frequently characterises the management of crises and disasters (Weaver, 1986; McGraw, 1990).

The characteristics described above could be very helpful, therefore, in understanding how local public leaders act when making crisis management decisions. Thus, depending on the different combinations of the four factors just listed and presented, we can expect local institutional leaders to be more or less likely to:

- take account of the early signals of a crisis, and hence apply the precautionary principle in the case of warnings from scientific institutions;
- prioritise between the different issues that arise during a crisis;
- interact and cooperate with local and national institutions;
- communicate in line with a leadership role, ensuring accurate sensemaking and meaning making;
- take autonomous decisions out of the routine tasks expected by formal roles; and
- be positively evaluated by the public.

Given the theoretical framework and expectations described so far, this paper analyses the leadership dynamics in the early crisis response activities of two Italian provinces affected by catastrophic flooding events in May 2023: Ravenna and Forlì‐Cesena.

## 3 RESEARCH DESIGN

### 3.1 The Emilia‐Romagna flood emergency

The two provinces of Ravenna and Forlì‐Cesena are part of the eastern subregion of Emilia‐Romagna Region in northern Italy, specifically named Romagna, and represent the two territories most severely affected by the flood events of 2023. They constitute the focus of the present research.

The two provinces share similar territorial characteristics (see the detailed description in Part 1 of the Supplementary Materials),^1^ yet operate under different political and administrative frameworks. The capital of Ravenna serves as a central hub for all administrative activities, whereas Forlì‐Cesena operates with two major urban centres (Forlì and Cesena), which function as co‐equal municipalities, each with its own separate administration and civil protection structures. In practice, this dual structure operates almost as two separate provinces housed within a single administrative box, potentially complicating emergency coordination.

**FIGURE 1:**
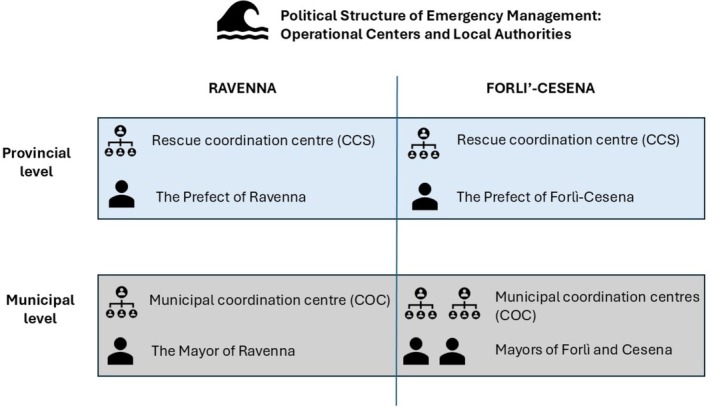
Political structure of emergency management: operational centres and local authorities in Ravenna and Forlì‐Cesena. source: authors.

During the two major flood events in this territory in 2023, the political structure of emergency management was organised at the provincial level, comprising two Rescue Coordination Centres (Centro Coordinamento Soccorsi, CCS) in Ravenna and Forlì‐Cesena, headed by two prefects, and three Municipal Operations Centres (Centro Operativo Comunale, COC): Ravenna, Forlì, and Cesena, led by the three mayors. Thus, we can consider the mayors and prefects to be the two most relevant local institutional leaders in the crisis management context (see Figure 1). More details regarding emergency management functioning as defined in the Italian Civil Protection Code are provided in the Supplementary Materials (Part 1).

In May 2023, the Italian region of Emilia‐Romagna was affected by two extreme flood events that occurred within just over two weeks of one another, specifically on 1–2 May and 16–17 May. The first event of moderate intensity was followed by a significantly more severe episode, which exacerbated the vulnerabilities exposed by the initial flood, causing widespread devastation across the region. Cumulatively, 23 rivers overflowed, approximately 60,000 landslides were recorded, and 123 municipalities were affected by inundation (ARPAE, 2023). The floods resulted in 17 fatalities, displaced nearly 10,000 individuals, and generated estimated economic losses of EUR 8.5 billion (ARPAE, 2023).

The magnitude of these events was unprecedented in the regional context. In fact, the return period of the recorded precipitation was estimated to exceed 500 years based on World Meteorological Organization standards. Hence, the May 2023 floods are considered to be among the most significant hydro‐meteorological disasters to affect Italy in recent decades (Cremonini et al., 2024).

### 3.2 Materials and methods

The comparative analysis adopted in this study provides a valuable framework for understanding differing approaches to flood emergency management in Ravenna and Forlì‐Cesena during May 2023. The research integrated and triangulated three main data sources (Denzin, 1970): local press coverage; municipal ordinances; and semi‐structured interviews with key decision‐makers. This triangulation enhanced the reliability of the findings and ensured a comprehensive understanding of the events, minimising potential bias from relying on a single source (Patton, 1999).

Each source played a distinct role. First, local newspaper coverage (see Table 2 in the Supplementary Materials) enabled a detailed reconstruction of the crisis timeline—from early warnings to emergency response—emphasising governance response, information flow, and public communication. Second, municipal ordinances traced institutional responses and political priorities, revealing how local authorities understood and reacted to the evolving situation. Other official sources, such as civil protection plans and weather bulletins, supported the broader research framework and helped to validate or challenge leaders' narratives (details are provided in Table 3 of the Supplementary Materials). Lastly, semi‐structured interviews provided first‐hand accounts of crisis management (see Table 4 in the Supplementary Materials). Guided by a standardised protocol, the interviews explored key themes, including event chronology, coordination challenges, trust in alerts, and leadership experience. Two types of interviewees were consulted. First, interviews with principal local political authorities involved in emergency response, namely, the Mayors of Ravenna, Forlì, and Cesena, and the Prefects of Ravenna and Forlì‐Cesena (see Figure 1), provided insights into comparative leadership styles and decision making across the two territories. These individuals are referred to as ‘political‐institutional leaders’ throughout the paper. Second, interviews with operational personnel who were most actively engaged during the emergencies (such as civil protection officers and firefighters) were essential for understanding the behaviours of the leaders and corroborating the accounts given by the authorities themselves. The interview transcripts were analysed in NVivo software using a codebook‐driven approach (see Table 5 in the Supplementary Materials) that combines deductive and inductive methods to align the emerging themes with the study's analytical framework.

## 4 RESULTS

In the following subsections, the flood events and related decision‐making dynamics in both areas are reconstructed to facilitate the identification of the leadership styles that emerged in the two territories and to make comparisons of them. Particular attention is given to leadership characteristics across several key dimensions: trust and reliability in early warnings; application of the precautionary principle; prioritisation of actions undertaken; communication strategies; inter‐institutional coordination; and the ability or willingness to implement autonomous and effective solutions. As such, we aim to evaluate the extent to which individual leadership influences the effectiveness of crisis response and to pinpoint the underlying factors that drive decisions made during emergencies.

### 4.1 Crisis management in Ravenna

The first meteorological alerts for the 2023 Emilia‐Romagna floods, indicating ‘ordinary’ (yellow) flood risk, were issued in late April by the Regional Civil Protection Agency and the Weather Alert Centre (Valente et al., 2025). In the province of Ravenna, the alert level rose rapidly to ‘moderate’ and then to ‘high’ by midday on 2 May. While the city of Ravenna remained under an orange alert, red alerts were issued unevenly across Lower Romagna, specifically in the areas that were later most affected by river overflows and levee failures.

The severity of the forecasts was taken seriously by the Prefect of Ravenna, who took immediate action by establishing the CCS on 2 May. Here, owing to persistent and worsening weather conditions, coupled with repeated severe weather warnings, the CCS remained continuously active for nearly two months. Additionally, the 18 COCs were also activated as precautionary measures in each of the municipalities within the province of Ravenna.

For the second flood (16–17 May), alert interventions began as early as Sunday, 14 May, when a red alert was issued by the Regional Civil Protection Agency, warning of an extremely high‐risk scenario starting on 16 May. The prefect decided initially to call an urgent meeting with all municipal administrators on Monday, 15 May, to share the alert with citizens and establish a unified response plan, as advised by regional experts. Ultimately, though, to avoid delay, the prefect opted to call all mayors for an urgent meeting on the Sunday afternoon (14 May). The early warning system initially received criticism, but local authorities now believe it saved countless lives given the massive scale of subsequent events. The mayor and the prefect used multiple channels—social media, police megaphones, and door‐to‐door outreach—to communicate evacuation instructions.

The efforts of the political‐institutional leaders responsible for managing emergencies were fully directed towards saving as many human lives as possible, even when new methods had to be devised to achieve this objective. Emergency protocols, in fact, do not obligatorily require preventive evacuations, since this measure is likely to be applicable *during* the unfolding of the event (Municipality of Ravenna, 2021). However, considering the severity of the meteorological alerts and the uncertainty surrounding crisis progression, anticipatory evacuations were enacted on the sunny day preceding the onset of rainfall (Ordinance No. 721, 16 May 2023), even in some at‐risk areas that did not experience direct flooding in the following hours or days. Overall, the evacuation of citizens from high‐risk areas represented one of the main, and most significant, measures adopted from 15 May, the day before the onset of heavy rainfall. In light of this timing aspect, the intervention must be regarded as an anticipatory measure prompt by precautionary attitudes. Following the prefect's recommendation, numerous mayors from the 18 municipalities issued preventive evacuation orders for the most at‐risk areas, primarily targeting houses or entire neighbourhoods near riverbanks. This approach was also adopted in the urban centre of Ravenna, where inhabitants were evacuated from areas near the Ronco and Montone Rivers, which converge near the city into what is known as the ‘Fiumi Uniti’ (Carloni, 2023).

A broad network of local authorities, professionals, and trade associations had to be coordinated to manage an emergency of this magnitude. Each contributor was responsible for key fundamental aspects disrupted by the event, including road infrastructure, rescue, displacement, healthcare, and waste management. The successful coordination in Ravenna demonstrates how leadership plays a vital part in coordinating stakeholders and keeping them united. Following the emergency, the municipal administration of Ravenna focused on evaluating the events and identifying potential shortcomings to demonstrate its commitment to internal and public accountability processes pertaining to the incident. The public generally viewed flood emergency management as positive. The 2024 regional elections backed up this perception when the mayor who became president of Emilia‐Romagna Region received 57 per cent of votes cast. During his presidential campaign, the national media consistently depicted him as the mayor who protected Ravenna from the flood, which solidified his reputation as an effective crisis leader.

### 4.2 Crisis management in Forlì‐Cesena

During the first flood event in the province of Forlì‐Cesena, the weather alert for river flooding remained constant at a moderate‐risk level (orange alert). Between 2 and 4 May, though, the territory experienced high criticality with respect to landslides, overflows, and flooding, the latter of which also occurred in the urban centre of Forlì owing to the overflow of the Montone River (Petrillo, 2023). Residential evacuations were limited to the hilly and foothill areas of the province in response to land erosion and landslides.

Nevertheless, no emergency coordination centres were active for the early May floods at the provincial or municipal level. In fact, the review of press coverage and official ordinances shows that the COC in Forlì was established only several days after the flood's most critical peaks. Indeed, the emergency coordination hub was established through the mayor's decree on 10 May and then remained in force to cover the subsequent flood events of 16–17 May. Consequently, during the initial critical phase, Forlì and its province lacked an active territorial command post capable of promptly coordinating all stakeholders involved in crisis response, despite much of its territory being inundated.

The delay in establishing a crisis coordination hub may be related to incomprehension or even underestimation of the severity of the meteorological alerts issued by the competent authorities. This was also evident in the lack of activation of preventive evacuation measures. Evacuations were issued in the city of Forlì during the critical phase of the second event and were formalised only during the emergency itself—namely, through Ordinance No. 14 of 17 May 2023—after flooding had already reached the neighbourhoods along the river. ‘We were not expecting such a disaster’ is what the Prefect of Forlì‐Cesena subsequently stated (in an interview on 14 November 2024). Thus, local political leaders appeared to comprehend fully the severity of the situation only as it unfolded, which resulted in a lack of anticipatory or precautionary action.

A significant element in the emergency governance structure emerged during the critical phases of the crisis, stemming from the absence of the primary civil protection officer for the municipality of Forlì. This absence was due to the officer himself being severely affected by the flooding, along with his family, in the nearby city of Faenza. The lack of this institutional expertise appears to have had serious consequences for the coordination of the initial disaster response, as the mayor was not supported by a qualified and competent figure in crisis management, who later turned out to be the only municipal official with adequate knowledge of emergency protocols and civil protection plans. This aspect, which played a central role in both the disputes within the administration and in media coverage during the post‐disaster phase, highlights significant structural weaknesses in the municipality's crisis preparedness. Furthermore, the mayor's leadership was inevitably undermined by this lack of support. This exposed weaknesses in the early detection of the disaster, the coordination of actors within the COC, and the prompt activation of preventive measures.

Despite these limitations, with regard to public disaster communication, both the mayors and the prefect in Forlì and Cesena activated mass public information systems through all available channels, including civil protection networks, private telecommunications, street megaphones, and social media. Notably, both mayors made extensive use of their personal social media profiles to disseminate timely updates, clarify emergency measures, and maintain direct contact with citizens.

For example, the post‐emergency deliberation process at Forlì's municipal council was marked by significant political conflicts. The opposition parties requested the establishment of a committee of inquiry on the flood emergency (Transparent Administration of the Municipality of Forlì, 2023) to study emergency response and detect possible weaknesses after the disaster. As required by Italian law (Legislative Decree No. 267 of 2000),^2^ the committee consisted of 14 members of the municipal council, whose composition was guided by the principle of proportional representation of council groups. From the outset, the establishment of this committee met with disagreement by the council's majority, who accused the proponents of the inquiry of engaging in political propaganda. This oppositional attitude was evident within the local political arena throughout the committee's operational period, which ran from August 2023 until April 2024. The repeated absences of members of the political majority, for instance, were highlighted in media coverage. This resulted in delays to sessions and obstruction of the committee's progress (Campanella, 2024).

The final inquiry report concluded that there were ‘no substantial failures’ by the administration (Campanella, 2024), a judgement contested by some members of the opposition, who called it ‘a clumsy attempt to distort reality’ (Bondi, 2024). Signed only by the committee's chair, the report was seen as unrepresentative of the committee's collective views. What was meant to be a constructive debriefing devolved into a political confrontation, limiting its operational relevance and hindering institutional learning. This outcome also provoked disillusionment among local neighbourhood committees and flood victim committees, which, upon the conclusion of the internal inquiry, highlighted serious failures in emergency management, described as ‘embarrassing’ (Bucchi, 2024).

### 4.3 Leaders in crisis: expectations and drivers of behaviours

Table 1 summarises the two cases previously described, focusing on how the role of leaders aligns with the six behavioural expectations outlined at the end of section two.

**TABLE 1:** Comparison between dimensions/expectations in relation to leadership capacities: Ravenna and Forlì‐Cesena.

Dimensions/expectations	Ravenna	Forlì‐Cesena
**Consideration of the early signals**	Forecasts and alerts were taken seriously, and leaders promptly reacted with (i) activation of emergency coordination centres and (ii) preventive evacuations.	Underestimation of the alerts and delay in establishing a crisis coordination hub. Evacuation measures were activated during events.
2 **Prioritisation**	To ensure the safety of the population, including through measures not strictly mandated by official protocols.	To adhere strictly to protocols and emergency plans.
3 **Cooperation and coordination**	Broad network of local authorities and professionals positively cooperated throughout all aspects of emergency management. The structure of crisis governance ensured autonomy within coordination.	Both during the emergency phase and post disaster, the level of cohesion and coordination among the involved actors was difficult to ensure (a situation compounded by the forced absence of the civil protection officer).
4 **Communication**	Mass risk communication was activated through all available channels.	Mass risk communication was activated through all available channels.
5 **Autonomous decisions out of the routine tasks**	Protocols and emergency plans were adapted to the context and leaders acted in conformity with the changing character of the events.	Strict adherence to protocols prevented the adoption of creative and adaptive behaviours.
6 **Positive evaluations by the public**	Overall positive public evaluation of emergency governance, as demonstrated by the election of the mayor to the presidency of the region.	Political tensions within the deliberation process internal to the administrative council ended with the establishment of a ‘council committee of inquiry’ pertaining to flood management. Dismay among local citizens' committees.

**Source:** authors.

The difference between the two cases is clear and distinct and can be explained by the key factors that influence local leaders' behaviour, as outlined in section one and identified in the literature: contextual factors; prior experiences; epistemic orientation; and decision‐making style. With respect to contextual factors, the two provinces differed structurally in one respect: Forlì‐Cesena's dual‐city structure operates effectively as two separate municipalities within one province, which creates potentially more complex coordination demands than Ravenna's unified structure. Yet, both provinces experienced the same extreme weather events and shared a centralised national civil protection system. They also had emergency plans that were updated simultaneously in 2021. Additionally, while the two territories differed in political orientation—Ravenna was governed by a centre‐left administration whereas Forlì‐Cesena had a centre‐right leadership—this does not appear to have systematically influenced crisis response patterns in ways that align with ideological expectations (Boin, Kuipers, and Overdijk, 2013; Christensen, Lægreid, and Rykkja, 2016). The critical question is whether the structural coordination difference or political orientation explains the observed leadership outcomes. Our evidence suggests that neither does. The coordination challenge in Forlì‐Cesena was manageable, but it was poorly navigated owing to leadership factors, particularly the absence of a civil protection officer and the delayed response of leaders to warnings. Therefore, it is necessary to shift the analytical focus towards other explanatory dimensions rooted in individual leadership characteristics.

As noted, experience of prior emergencies can significantly impact how local leaders respond under pressure. The ability to identify, attend to, and interpret ambiguous and unfamiliar signals characteristic of crisis situations is often regarded as a core leadership competency that is closely linked to previous experience of crisis management. In this respect, the command of the flood emergency response in Ravenna was led by a prefect whose prior experience as deputy prefect in one of the main cities affected by the 2016–17 central Italy earthquake—one of the most significant disasters in recent Italian history—proved instrumental to his leadership role.

The Mayor of Ravenna emphasised in his interviews that political capacity and emergency experience directly correlated during the 2023 floods, as different levels of experience among political authorities yielded varying emergency governance results. He remembered that several colleagues had recently taken office and did not possess the necessary crisis response skills or equipment. The institutional leaders of Forlì‐Cesena appeared to have more limited exposure to emergency management. The Prefect of Forlì‐Cesena, for example, recalls managing two heavy snowfalls during a previous mandate in two central Italian cities (Ancona and Chieti). Similarly, the mayor referred to his previous experience of managing a snowfall during his tenure as the mayor of a smaller municipality in Romagna (Meldola). However, these events were primarily of local significance and did not leave a notable mark on the country's collective memory.

In discussing leaders' capacity to orchestrate coordination among stakeholders involved in crisis management, for instance, one of the Ravenna institutional leaders remarked that ‘the role of coordination becomes a profession—it is a technical skill that one acquires either through experience or, ideally, through specific training courses. … I am firmly in favour of instituting compulsory trainings for mayors in the field of civil protection’. It was also emphasised that severe weather alerts during his eight‐year term, although not resulting in actual flooding, had helped him to understand better the emergency measures to be implemented, as well as to carry out emergency training practices.

In contrast, in Forlì, the absence of the official responsible for civil protection during the most critical phases of the crisis exposed major structural weaknesses in local governance, not at all compensated by the leadership's capacity on the ground. The interview data revealed that the limited civil protection expertise acquired by local leaders in ‘peacetime’ became particularly evident in the management of the May 2023 emergency, especially with respect to orchestration and coordination of the actors involved in the emergency hub.

Local leaders' reliance on scientific expertise—and their ability to incorporate technical data and risk forecasts into decision making—can provide valuable insights into their epistemic orientation. In Ravenna, institutional leaders demonstrated a high degree of trust in meteorological alerts from the outset of the flood events. As recalled by one, on 14 May, forecasts began to signal a ‘catastrophic alert, a cyclonic event expected to strike Romagna, requiring prompt and pre‐emptive action’. This triggered an immediate local response, including the activation of emergency coordination hubs and the implementation of preventive evacuation measures. The critical hours following the forecasts are accounted for in detail in the following remarks of a Ravenna institutional leader:

*It was a sunny Sunday when I called all the mayors together, and I had to convince them that after the first emergency, an even more severe one was on its way. The mayors were somewhat incredulous, saying, ‘it's sunny today, who among the citizens is going to trust this alert?’*

*From the moment we received that information, the emergency response system was activated right away, and I must say: that was what saved us. All the mayors recognise it now: if we hadn't met that Sunday and the information hadn't begun circulating among the population from that point on, we would have seen hundreds of deaths, because what followed was truly devastating*.

The attitudes of the leaders in Forlì‐Cesena with regard to the risk forecasts appeared to be particularly different, such as being characterised by prudence and a scarce level of trust in the alerts, as demonstrated by the words of the Prefect of Forlì‐Cesena: ‘We knew there would be severe weather, but we did not imagine a disaster of that magnitude. We can predict that perturbations will arrive, but not their precise effects. … It is not a mathematical certainty that a flood will occur’.

Despite the availability of predictive data indicating severe atmospheric disturbances, the failure to anticipate the true scale of the disaster reflects a gap between probabilistic information and anticipatory action, whereby such information failed to trigger preventive measures. In fact, decision‐makers in Forlì‐Cesena seemed reluctant to enable responsiveness until the crisis had already materialised.

This varying degree of trust in meteorological alerts was concretely reflected in the timing of decisions made during the early hours of the emergency. In Ravenna, provincial and municipal emergency coordination centres were promptly activated, whereas in Forlì, the COC was established only in response to the impacts of the floods in early May. Similarly, in Forlì‐Cesena, evacuation orders were issued only once the severity of the crisis had become evident, whereas in Ravenna, local leaders opted to implement anticipatory evacuation measures. Thus, leaders who place trust in alerts are likely to make bold and timely decisions—even when those choices may be unpopular—because they believe that protective actions, although potentially contested in the short term, can ultimately save lives. According to the Mayor of Ravenna:

*There was a genuine risk of significant loss of life had we not proceeded with evacuations. In that context, it was worth it even if citizens, in hindsight, felt it was unnecessary and complained, maybe saying: ‘they made us leave our homes for nothing; we spent a night on a camp bed for nothing’. As a mayor, these are the difficult decisions one is required to make*.

With respect to decision‐making style, leaders in Ravenna demonstrated proactive behaviour not only through their prompt response during the initial stages of the flooding, but also through the active engagement of the municipal administration in the post‐emergency debriefing process, showing a clear commitment to identifying shortcomings and addressing them. As one of the Ravenna institutional leaders stated: ‘I am a strong advocate of debriefing … the work we carried out in the following months focused on thoroughly reviewing every minor imperfection or aspect that could have been handled better’, highlighting a concern for both an internal and public process of accountability regarding the events. In contrast, in Forlì, the absence of a learning‐oriented approach became evident through the collapse of the internal evaluation process following the flooding. Rather than fostering reflection and reform, the process revealed entrenched divisions and an inability to translate crisis into constructive learning.

An additional dimension of a proactive decision‐making style lies in the capacity to adopt a precautionary approach. Anticipatory action involves adaptive problem solving and flexible decision making, particularly when established protocols prove inadequate in dynamic or uncertain contexts. This approach was exemplified in Ravenna, where authorities issued evacuation ordinances pre‐emptively (yellow alert), implementing a measure that, according to the municipality's civil protection plan, is typically reserved for a later stage in crisis progression.^3^

By anticipating evolving risks, local authorities in Ravenna demonstrated the ability to move beyond procedural rigidity. They responded with situational awareness and strategic foresight, accepting the risk of being criticised if the evacuation was not useful. Furthermore, creative solutions were found to guarantee shelter for all residents living in high‐risk areas: ‘We invented everything we could: we turned a cinema into a shelter, a museum into a hub, since the locations designated in the emergency protocols were no longer sufficient’.

When examining the relationship between decision‐making style and emergency protocols, noteworthy reflections emerged from the leaders interviewed in the province of Forlì‐Cesena. For example, Forlì institutional leaders devoted a significant portion of the interviews to addressing the constraints posed by existing emergency management protocols: on several occasions, the municipal administration reported feeling hindered by the limitations imposed by the bureaucratic apparatus and by the structural framework through which crisis governance is distributed. For instance: ‘We were in a situation where we didn't know how many people had died, nor how many families needed to be brought to safety, and I was expected to study bureaucracy—this is something inconceivable. … This means losing crucial opportunities for action in the first few hours, which are the most important for ensuring the safety of people and property’.

In addition to the limited previous knowledge of emergency procedures, these words demonstrate Forlì's strong adherence to official protocols (‘the protocols are essentially defensive in nature’). Certain decisions that were not fully aligned with emergency plans—described by the Mayor of Forlì as ‘borderlines’—were seen as potentially exposing political decision‐makers to ‘legally slippery and risky situations’. This attitude suggests a crisis management approach primarily oriented towards passive compliance, with the aim of avoiding criticism or future repercussions: a potentially mistaken decision, if made in accordance with established rules, is generally more defensible and legitimate.

Overall, decision‐making styles in the contexts under examination can be described as proactive and creative in Ravenna and routine and defensive in Forlì‐Cesena. Table 2 summarises the differences in the drivers of leaders' behaviour.

**TABLE 2:** Comparison of the drivers of leaders' behaviour: Ravenna and Forlì‐Cesena.

Drivers	Ravenna	Forlì‐Cesena
**Contextual factors**	Centralised civil protection governance structure. Local autonomy within coordination. Recent update of civil protection plans. Severe and high‐risk emergency context.	Centralised civil protection governance structure. Local autonomy within coordination. Recent update of civil protection plans. Severe and high‐risk emergency context.
2 **Prior experiences**	High level	Medium to low level
3 **Epistemic orientation**	Strong reliability of alerts and forecasts. High degree of trust in scientific expertise.	Low reliability of alerts and forecasts. Low degree of trust in scientific expertise.
4 **Decision‐making style**	Proactive: readiness of response and activation of preventive measures. Post‐disaster learning‐oriented approach. Creative action and adaptive approach to crisis management.	Routinary and passive: delay in response and reactive activation of preventive measures. Defensive post‐disaster approach towards debriefing. Strong adherence to pro emergency plans.

**Source:** authors.

## 5 DISCUSSION

The comparison between Ravenna and Forlì‐Cesena highlights the critical role that individual leadership behaviour plays in shaping the trajectory and outcomes of local crisis management (Boin, Kuipers, and Overdijk, 2013). The presence of highly similar institutional structures, legal frameworks, and emergency protocols in both provinces reinforces the conclusion that leadership agency, rather than structural differences, was the decisive factor influencing the effectiveness of the crisis response. This underlines the importance of crisis management as a dynamic, interpretive, and often high‐stakes endeavour that is heavily influenced by the qualities and orientations of the individuals leading it.

The Ravenna case illustrates the outcomes that can be achieved when leaders utilise their discretionary space with a sense of responsibility, urgency, and adaptability (Boin and Lodge, 2016). The prior crisis experience, epistemic practices for engaging with scientific forecasts, and capacity for decisive action under uncertainty demonstrated by Ravenna leaders did not eliminate uncertainty, but these factors helped to convert that uncertainty into a space for protective action. This approach shows how proactive, context‐sensitive local leadership can absorb institutional complexity and transform it into a source of coordinated, meaningful action (Ansell, Sørensen, and Torfing, 2021).

In contrast, the case of Forlì‐Cesena reveals what happens when leadership defaults to a rule‐bound and risk‐averse mode in the face of crisis. Here, decision‐makers seemed reluctant to take decisive action until the flooding had already materialised. Their reliance on procedural legitimacy over substantive responsiveness constrained their ability to take pre‐emptive action. The potential for flexible and timely intervention was hindered by reluctance to deviate from formal emergency plans, even when they proved inadequate for the unfolding situation. While this routine behaviour may have been justified in bureaucratic terms, it was ultimately misaligned with the demands of a fast‐evolving disaster (Comfort, 2007; Moynihan, 2009).

Crucially, these leadership differences cannot be attributed to political party dynamics or coalition pressures alone. Although the two provinces had different political orientations—Ravenna was governed by a centre‐left administration whereas Forlì‐Cesena was governed by a centre‐right coalition—this ideological distinction does not systematically explain crisis response patterns. Research on disaster governance shows that when it comes to rapid‐onset emergencies, political ideology often proves less consequential than organisational capacity, prior crisis experiences, and leaders' epistemic practices (Comfort, 2007; Slavíková, Raška, and Kopáček, 2019). The divergence reflects how individual leaders, shaped by their experiences and epistemic orientations, interpreted their roles within broadly similar institutional and political constraints. This is consistent with studies showing that crisis situations often transcend routine political divisions, rendering leadership competencies more important than party affiliation (Boin, Kuipers, and Overdijk, 2013; Christensen, Lægreid, and Rykkja, 2016). Scholarship on scientific advice and political decision making confirms that the same expert forecast can trigger different responses depending on institutional practices for managing uncertainty rather than political positioning (Braun and Kropp, 2010).

Moreover, the contrast extends beyond the initial emergency response to the post‐crisis phase, as institutional leaders' behaviour continued to diverge. In Ravenna, the initiation of a systematic debriefing process signalled a commitment to institutional learning, public accountability, and continuous improvement. The leaders there appeared willing to scrutinise their own actions, acknowledge imperfections, and use the disaster as a catalyst for strengthening their governance capabilities. This is a key characteristic of effective leadership: not only managing the crisis itself but also extracting learning from it and reinvesting that learning in the community's future preparedness (Boin et al., 2005).

In Forlì‐Cesena, the post‐crisis phase diverged sharply from this learning‐oriented approach, and exposed a very different orientation. The internal evaluation process suffered from a deficiency in a learning‐oriented mindset, which became apparent in the council committee of inquiry established to investigate flood management. The investigation evolved into a political dispute, which damaged both the legitimacy and effectiveness of the process. The gap between official institutional stories and the real‐life experiences of affected communities intensified public frustration, weakening trust in local leadership. This breakdown in meaning making and accountability represents a missed opportunity for post‐crisis repair and rebuilding of public trust (’t Hart, 2014).

This comparison has clear and far‐reaching implications. Effective local crisis leadership is not simply a function of institutional capacity or the robustness of formal procedures; it is fundamentally about the behaviour of leaders within, around, and beyond those institutional boundaries. While legislation, protocols, and emergency plans provide a framework, they cannot substitute for good judgement, courage, and responsiveness. The capacity to recognise risk early, especially when signals are ambiguous or contested, to act on imperfect yet credible information, to exercise discretion with integrity, and to guide collective action through periods of uncertainty and fear are not just technical proficiencies or innate psychological traits; rather, they are capabilities developed through experience, training, and organisational practices that can be strengthened by systematic leadership development.

This empirical analysis makes three contributions to disaster scholarship. First, by comparing two provinces with highly similar institutional frameworks but different outcomes, we have identified leadership as the decisive explanatory factor. This design addresses a persistent gap in the literature: while scholars recognise that individual capacities and structural conditions matter (Comfort, 2007; Boin and Lodge, 2016), few studies have systematically examined their relative influence. Our findings demonstrate that when structural variables are constant, it is individual leadership characteristics—prior experience, epistemic orientation, and decision‐making style—that become determinative.

Second, we reconceptualise epistemic orientation as an institutionalised practice rather than a psychological trait. While the literature acknowledges that leaders must engage with scientific advice in situations of uncertainty (Christensen and Lægreid, 2020; Ansell, Sørensen, and Torfing, 2021), it rarely specifies the behavioural aspects of this. Our analysis reveals concrete practices, such as establishing consultation mechanisms, communicating uncertainty transparently, and developing organisational capacity to act on probabilistic information. Crucially, prior crisis experience shapes these practices: leaders develop ‘practised uncertainty tolerance’ through repeated high‐stakes decision making. This moves beyond the concept of ‘evidence‐based decision making’ to encompass learnable, transferable capabilities.

Third, we extend the temporal analysis to include post‐crisis learning and accountability, not just emergency response. Although disaster research examines response effectiveness extensively, the link between crisis‐phase leadership and post‐crisis institutional behaviour remains underexplored.

Together, these contributions shift disaster leadership scholarship away from descriptive accounts of ‘success’ and ‘failure’ towards the analytical specification of the behavioural drivers that distinguish crisis management approaches.

## 6 CONCLUSION

The crisis behaviour of local leaders is influenced by interconnected drivers. Context defines constraints and opportunities, but it does not structurally determine agency. Individual experience provides confidence and insight, while trust in science ensures evidence‐based decision making. Leadership style, meanwhile, mediates communication and collaboration. Understanding these factors is essential for effective local crisis governance. This study identifies three priorities for strengthening it:

- First, leadership development should move beyond procedural knowledge and legal compliance, encompassing emotional intelligence, communication skills, and the ability to coordinate under pressure (Eid et al., 2023). Leaders must remain composed in uncertain situations, inspire confidence in stakeholders, and make informed, timely decisions.
- Second, epistemic foundations require increased attention. Leaders must engage effectively with expert knowledge, scientific data, and risk assessments. Crisis response demands technical competence and the political courage to act on uncertain forecasts while maintaining transparency and public trust. This necessitates critical inquiry, reflective thinking, and revising strategies based on new evidence.
- Third, accountability systems and organisational practices should encourage calculated initiative and adaptive problem solving in uncertain situations. Overly formalised systems produce risk‐averse leaders who fear acting outside of their defined authority. Accountability systems must support good‐faith discretion, distinguishing defensible improvisation from negligence. Leaders must adapt protocols to evolving circumstances, moving beyond defensive compliance towards responsible initiative.

Ultimately, outcomes depend not only on institutional frameworks, but also on how leaders interpret their roles, manage uncertainty, and exercise their judgement. In high‐stakes situations, it is human agency, not procedures, that determines whether resilience or dysfunction prevails. It is essential to foster technically competent and ethically grounded local leadership to navigate increasingly frequent and complex contemporary crises.

## CONFLICT OF INTEREST STATEMENT

The authors declare that they have no known competing financial interests or personal relationships that could have appeared to influence the work reported in this paper.

## FUNDING STATEMENT

This study was carried out within the RETURN Extended Partnership and received funding from the European Union's Next‐Generation EU instrument (National Recovery and Resilience Plan – NRRP, Mission 4, Component 2, Investment 1.3 – D.D. 1243 2/8/2022, PE0000005).

## Supporting information


**Figure 1** MAPS OF THE GEOGRAPHICAL CONTEXT: ON THE LEFT: THE EMILIA‐ROMAGNA REGION. ON THE RIGHT: THE PROVINCES OF RAVENNA AND FORLI’‐CESENA.
(SOURCE: AUTHORS' CREATION)
Figure 2. Scheme of the structural organisation of the National Civil Protection Service (authors' creation, source: Articles n.8, n.9, n.11, n.12, Section II ‐ Italian Civil Protection Code. Legislative Decree No. 1 of 2 January 2018.)
Table 2. Structural organisation of the National Civil Protection Service (authors' creation, source: Articles n.8, n.9, n.11, n.12, Section II ‐ Italian Civil Protection Code. Legislative Decree No. 1 of 2 January 2018.)
Table 2. Main sources for the press review
Table 3. official documents
Table 4. list of interviews
Table 5. codebook

## Data Availability

The data that support the findings of this study are available on request from the corresponding author. The data are not publicly available due to privacy or ethical restrictions.

## Endnotes

^1^
All supplementary materials can be found online in the supporting information section at the end of the article.

^2^
Legislative Decree No. 267 of 18 August 2000, ‘Consolidated Law on the Organization of Local Authorities (TUEL)’, Art. 38.

^3^
Specifically, Ravenna's *Municipal Civil Protection Plan* (Municipality of Ravenna, 2021, p. 344) stipulates that procedures for the possible evacuation of people at risk are to be activated starting from the orange alert (medium risk) and during the ongoing event, whereas the *Intermunicipal Civil Protection Plan of Romagna Forlivese* (Union of Municipalities of the Romagna Forlivese Area, 2021, p. 8), which includes the municipality of Forlì, states that the mayor, if necessary, can issue an evacuation order for homes at risk during the red alert phase (high risk).
