# Supplementary material for: Local responses to the threats of dramatic crises: do institutional leaders make a difference, and if so, how?
Source: Disasters. 2026 May 15;50:e70059. doi: 10.1111/disa.70059 (PMC13179474; doi:10.1111/disa.70059)
Supplement: Supplementary file 1 — Figure 1 MAPS OF THE GEOGRAPHICAL CONTEXT: ON THE LEFT: THE EMILIA‐ROMAGNA REGION. ON THE RIGHT: THE PROVINCES OF RAVENNA AND FORLI’‐CESENA. (SOURCE: AUTHORS' CREATION) Figure 2. Scheme of the structural organisation of the National Civil Protection Service (authors' creation, source: Articles n.8, n.9, n.11, n.12, Section II ‐ Italian Civil Protection Code. Legislative Decree No. 1 of 2 January 2018.) Table 2. Structural organisation of the National Civil Protection Service (authors' creation, source: Articles n.8, n.9, n.11, n.12, Section II ‐ Italian Civil Protection Code. Legislative Decree No. 1 of 2 January 2018.) Table 2. Main sources for the press review Table 3. official documents Table 4. list of interviews Table 5. codebook [file DISA-50-e70059-s001.docx]

**SUPPLEMENTARY MATERIALS**

**PART 1. Territorial and institutional aspects of the research context**

The provinces of Ravenna and Forlì-Cesena are both located in the Romagna area of Italy’s Emilia-Romagna region and represent the two territories mostly impacted by the 2023 flooding events. The province of Ravenna covers an area of approximately 1,859 km² and has a population of around 384,000 inhabitants. The province of Forlì-Cesena spans about 2,378 km² and is home to roughly 394,000 people. The geo-morphological and territorial configuration of the two provinces is notably similar, as both encompass a mountainous and hilly section belonging to the Apennine range, a broad urbanized and industrial plain, and a coastal area overlooking the Adriatic Sea. The three main river courses that cross both the provinces, and which were interested by overflows during the 2023 floods, are the Montone, Ronco and Savio.

Figure 1. MAPS OF THE GEOGRAPHICAL CONTEXT: ON THE LEFT: THE EMILIA-ROMAGNA REGION. ON THE RIGHT: THE PROVINCES OF RAVENNA AND FORLI’-CESENA.

(SOURCE: AUTHORS’ CREATION)


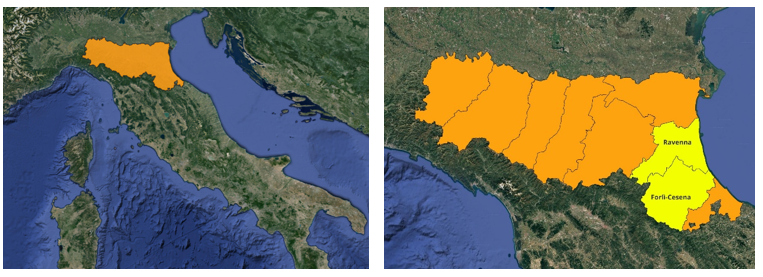


Despite their territorial similarities, the provinces of Ravenna and Forlì-Cesena differ in political and administrative structure. Ravenna has a centralized system, with a single capital serving as the hub for all functions. In contrast, Forlì-Cesena features two major urban centres, which share population size, services, and institutional roles. This dual structure reflects a long-standing functional balance and shared leadership but also introduces more complex coordination dynamics due to the distribution of key infrastructures and responsibilities across both cities.

In the Italian Civil Protection System, provinces and municipalities play a central role in managing local emergencies: civil protection in Italy is structured across four levels (national, regional, provincial, and municipal), all interconnected by the principle of subsidiarity, which ensures coordination while preserving the autonomy of each level (Dolce et al., 2020). The Civil Protection Code (Legislative Decree n.1 of 2 January, 2018) states that the principle of subsidiarity is divided into three fundamental dimensions. Firstly, *vertical subsidiarity*, according to which each higher level – provincial, regional or national – intervenes only when the lower level is no longer able to cope with the event using its own resources and skills, thus ensuring a gradual and consistent approach to emergency management. This is accompanied by the principle of *proportionality*, which ensures that the level of response is appropriate to the severity, complexity and territorial extent of the flood, avoiding both insufficient and excessive intervention. Finally, *loyal cooperation* between municipalities, regions and the state ensures constant and structured coordination, which is necessary to avoid operational overlaps, optimise the use of available resources and ensure effective and unified action during the emergency.

At a local level, the Italian Civil Protection System mandates that the coordination and management of crisis situations are conducted through local-level decision-making bodies comprising representatives from various operational functions. For instance, during emergencies, the Rescue Coordination Centre (*Centro Coordinamento Soccorsi*, CCS) is set up at the provincial level and led by the Prefect, a public official who heads the Territorial Office of the Government, whereas at the municipal level, the Municipal Operations Centre (*Centro Operativo Comunale*, COC) is established and led by the mayor.

Figure 2. Scheme of the structural organisation of the National Civil Protection Service (authors’ creation, source: Articles n.8, n.9, n.11, n.12, Section II - Italian Civil Protection Code. Legislative Decree No. 1 of 2 January 2018.)


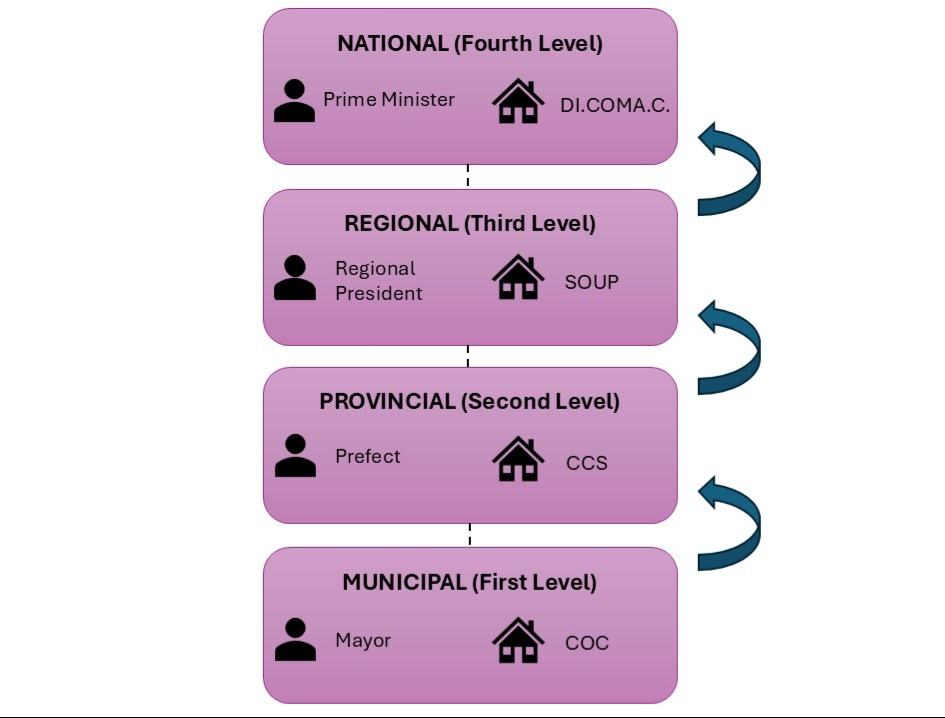


Table 2. Structural organisation of the National Civil Protection Service

(authors’ creation, source: Articles n.8, n.9, n.11, n.12, Section II - Italian Civil Protection Code. Legislative Decree No. 1 of 2 January 2018.)

| **Level** | **Responsible Authority** | **Structure to be activated** | **Main tasks** | **When does it intervene** |
| --- | --- | --- | --- | --- |
| **Municipal**  **(First level)** | Mayor | C.O.C – Municipal Operations Centre. | -First emergency response; -Public communication;  -Implementation of the municipal emergency plan;  -Evacuation orders;  -Coordination of local volunteers. | As soon as the event requires it. |
| **Provincial**  **(Second Level)** | Prefect | C.C.S – Provincial Coordination Center. | - Coordination of police forces and fire brigade - Support to affected municipalities - Management of mobility and public order | When the flood affects multiple municipalities or when one or more municipality require additional resources. |
| **Regional**  **(Third Level)** | Regional President | S.O.U.P – Regional Operations Room. | - Declaration of regional state of emergency - Coordination of regional response teams - Hydro-meteorological alert system - Request for national emergency | When the event exceeds provincial capacity or affects large territorial areas. |
| **National**  **(Fourth level)** | Prime Minister’s Office / Civil Protection Department | DI.COMA.C. - Command and Control Department. | - Declaration of national state of emergency - Coordination of national operational structures - Mobilisation of extraordinary resources - Emergency ordinances | When the event exceeds regional capacity and/or extraordinary powers are required. |

**PART 2. Methodology and research materials**

The triangulation of sources enabled to strengthen the robustness of the provided information and to minimize the risk of distortions or biases inherent in relying on a single source of data. For instance, each of the three sources (local press, official documents and interviews) employed proved to be indispensable, each for distinct reasons.

First, the review of the local newspaper press (see Table 2 for sources’ details) enabled to reconstruct the events in chronological order: being timing a core factor in disaster occurrence and in crisis management, the reconstruction of the phenomenon in its unfolding – from the early alerts to the crisis response – is crucial for a comprehensive understanding of the events. Moreover, the real-time news coverage and its hour-by-hour and day-by-day evolution not only allow for an accurate reconstruction of events but also provide insight into the volume of information available – or, conversely, into its absence or inconsistency of information – during moments of uncertainty such as those triggered by crisis situations. Even more, this source of data makes it possible to understand what kind of information the public was exposed to in real time, thereby allowing for a better grasp of certain nuances in disaster communication.

Table 2. Main sources for the press review

| **Online press** | **Territorial coverage** | **Link** | **Timeframe** |
| --- | --- | --- | --- |
| RavennaToday | Ravenna and province | [https://www.ravennatoday.it](https://www.ravennatoday.it/) | 1^st^ May – 31^st^ May |
| RavennaNotizie | Ravenna and province | <https://www.ravennanotizie.it> | 1^st^ May – 31^st^ May |
| ForlìToday | Forlì and FC province | <https://www.forlitoday.it/> | 1^st^ May – 31^st^ May |
| CesenaToday | Cesena and FC province | <https://www.cesenatoday.it> | 1^st^ May – 31^st^ May |

The collection and analysis of official documents (Table 3) constituted a key source for reconstructing the events, with specific attention to political decision-making and the operational measures enacted by institutional command structures. This type of source played a dual role in the research’s analytical process: on the one hand, municipal ordinances offer important insights into the responsiveness of political decision-makers, both in terms of the timing of their reactions and the substance of the measures enacted. As such, they provide an indirect indication of how leaders perceived the severity of the events and how they were able to react. Hence, reliance on official documentation enabled the validation, or the questioning, of the course of events retraced by the interviewed political leaders. Further official documents – such as Civil Protection Plans and weather alert bulletins – were repeatedly consulted and served as secondary sources in shaping the research design.

Table 3. official documents

| **Document type** | **Territory** | **Link** | **Notes** |
| --- | --- | --- | --- |
| Evacuation ordinances | Ravenna and province | <https://www.comune.ra.it/alluvione-ed-eventi-meteoclimatici-avversi-del-2023/ordinanze/> | List of evacuation ordinances. |
| Flood emergency section of Ravenna’s province website | Ravenna and province | [https://www.provincia.ra.it/Argomenti/Speciali/Emergenza-alluvione#](https://www.provincia.ra.it/Argomenti/Speciali/Emergenza-alluvione) | Fully dedicated to news and in-depth coverage of the severe weather that struck the provincial territory. |
| Municipal ordinances issued for flood events | Forlì and province | <https://www.comune.forli.fc.it/servizi/Menu/dinamica.aspx?idSezione=8882&idArea=312876&idCat=312900&ID=312900&TipoElemento=categoria> | List of municipal ordinances regarding mobility restrictions, school closures, evacuations and relocations. |
| Civil Protection Plan – Municipality of Ravenna | Ravenna | <https://www.comune.ra.it/aree-tematiche/protezione-civile/piano-di-protezione-civile-comunale/> |  |
| Civil Protection Plan – Municipality of Forlì | Forlì | <https://www.comune.forli.fc.it/servizi/Menu/dinamica.aspx?idSezione=72535&idArea=145260&idCat=293213&ID=293213&TipoElemento=categoria> | Inter-municipal civil protection plan: ‘Romagna Forlivese Union of Municipalities. |
| Civil Protection Plan – Municipality of Cesena | Cesena, Bagno di Romagna, Mercato Saraceno, Montiano, Sarsina, Verghereto | <https://www.unionevallesavio.it/piano-intercomunale-cesena> | Inter-municipal civil protection plan: ‘Valle del Savio’ Union of Municipalities. |

Finally, semi-structured interviews were conducted with the leaders who, during the emergency, held authority over emergency management and civil protection operations (see Table 4). The interview guide submitted to the interviewees followed the same structure and list of questions, in order to enable a comparative analysis of the collected responses. In accordance with the theoretical framework and to the hypothesis that guided the research, the following topics were addressed: retrace of the events and critical aspects of crisis management; past experiences in disaster management; reliability/trust in meteorological alerts; considerations of inter-institutional coordination and timing. The ensemble of such information, put together, enabled to picture the leadership behaviour and decision-making style of the involved political actors.

Table 4. list of interviews

| **Political figure** | | **Administrative level** | **Interview date** |
| --- | --- | --- | --- |
| 1 | Prefect of Ravenna | Provincial (Ravenna) | 23^rd^ July 2024 |
| 2 | Mayor of Ravenna | Municipal (Ravenna) | 26^th^ August 2024 |
| 3 | Prefect of Forlì-Cesena | Provincial (Forlì-Cesena) | 14^th^ November 2024 |
| 4 | Mayor of Cesena | Municipal (Cesena) | 14^th^ January 2025 |
| 5 | Mayor of Forlì | Municipal (Forlì) | 5^th^ February 2025 |
| **Operational figure** | | **Administrative level** | **Interview date** |
| 6 | Civil Protection Services | Municipal (Ravenna) | 10^th^ May 2024 |
| 7 | Civil Protection Services | Provincial (Ravenna) | 6^th^ June 2024 |
| 8 | Fire Brigades | Provincial (Ravenna) | 7^th^ June 2024 |
| 9 | Civil Protection Services | Municipal and inter-municipal (Forlì) | 27^th^ May 2025 |

Interviews were transcribed verbatim, and a content analysis was conducted using NVivo qualitative analysis software. A codebook was developed based on the study’s objectives and theoretical framework and applied to the texts in order to deductively code the transcripts. At the same time, the coding process remained flexible, allowing for the inductive inclusion of new codes emerging from the data.

Table 5. codebook

| **Type of code selection** | **Code** | **Definition** | **Notes** |
| --- | --- | --- | --- |
| Codes selected **deductively** | Trust in early-warnings | The extent to which decision-makers consider official alerts from scientific or technical institutions to be credible and accurate, influencing their willingness to take precautionary measures before a crisis fully unfolds. |  |
|  | Precautionary Attitudes | The tendency of decision-makers to take early, preventive action in response to potential risks, even in the absence of full certainty. |  |
|  | Coordination and cohesion | The degree to which actors work collaboratively, share information, and align their acctions effectively during crisis management, ensuring a unified and efficient response |  |
|  | Prior experiences of crisis management | Previous involvement in managing emergency, which can influence their confidence, judgement, and effectiveness in responding to new crises. |  |
|  | Autonomous decisions | Actions or choices made independently by local leaders, based on their own assessment of the situation. |  |
|  | Leadership capacities | The abilities and qualities of individuals in leadership roles to guide, make decisions, and mobilize resources and actors, influencing the overall effectiveness of the emergency response. |  |
| Codes selected **inductively** | Evacuations | Local leaders’ actions and approaches towards preventive evacuation measures. | As sub-topic of “precautionary attitudes” |
|  | Creative action vs Adherence to protocols | The balance between flexible, context-specific solutions and strictly following established procedures and official guidelines in managing a crisis. | As sub-topic of “autonomous decisions” |

**References**

Dipartimento della Protezione Civile. (2018). *Codice della protezione civile* (D.Lgs. 2 gennaio 2018, n. 1). Gazzetta Ufficiale, n. 10, 15 gennaio 2018. Available at: <https://www.protezionecivile.gov.it/it/normativa/decreto-legislativo-n-1-del-2-gennaio-2018--codice-della-protezione-civile/> , last seen 15.12.2025.

Dolce M., Miozzo A., Di Bucci D., Alessandrini L., Bastia S., Bertuccioli P., Bilotta D., Ciolli S., De Siervo G., Fabi D., Madeo L., Panunzi E., Silvestri V. (2020). *La protezione civile in Italia. Testo istituzionale di riferimento per i docenti scolastici.* Dipartimento della Protezione Civile-Presidenza del Consiglio dei Ministri. Prima edizione luglio 2020.

Legislative Decree No. 1 (2 January 2018). *Civil Protection Code*. Official Gazette of the Italian Republic. Available at: <https://www.protezionecivile.gov.it/it/normativa/decreto-legislativo-n-1-del-2-gennaio-2018--codice-della-protezione-civile/>, last seen 12.12.25.
